# Humoral and cell-mediated immune responses in HIV-vertically infected young patients after three doses of the BNT162b2 mRNA SARS-CoV-2 vaccine

**DOI:** 10.3389/fimmu.2023.1301766

**Published:** 2024-01-04

**Authors:** Claudia Vanetti, Marta Stracuzzi, Elisa Crivellaro, Federica Ciciliano, Micaela Garziano, Claudio Fenizia, Mara Biasin, Valeria Rubinacci, Antonella Amendola, Elisabetta Tanzi, Gian Vincenzo Zuccotti, Mario Clerici, Vania Giacomet, Daria Trabattoni

**Affiliations:** ^1^ Department of Biomedical and Clinical Sciences, University of Milan, Milan, Italy; ^2^ Pediatric Infectious Disease Unit, Ospedale L. Sacco, University of Milan, Milan, Italy; ^3^ Department of Pathophysiology and Transplantation, University of Milan, Milan, Italy; ^4^ Department of Health Sciences, University of Milan, Milan, Italy; ^5^ Department of Pediatrics, Ospedale dei Bambini V. Buzzi, Milan, Italy; ^6^ IRCCS Fondazione Don Carlo Gnocchi, Milan, Italy

**Keywords:** PLWH, SARS-CoV-2 vaccine efficacy, immunocompromised subjects, COVID-19 SIV individuals, immune response

## Abstract

**Background:**

Data on the efficacy of three SARS-CoV-2 mRNA BNT162b2 vaccine doses and the role of previous SARS-CoV-2-infection in enhancing vaccine immunogenicity in HIV-vertically-infected people living with HIV (PLWH) are limited, as is the duration of vaccine-induced responses.

**Methods:**

SARS-CoV-2 plasma neutralizing activity (NA) against the European (B.1), Delta (B.1.617.2) and Omicron (B.1.1.529) variants and cell-mediated immunity (CMI) were analyzed in 29 ART-treated young PLWH (mean age 27.9 years) and 30 healthy controls (HC) who received three BNT162b2 vaccine doses. Individuals were stratified based on the presence/absence of previous SARS-CoV-2 infection (infected and vaccinated -SIV-; uninfected and vaccinated -SV-). Analyses were performed before vaccination (T0), 25 days from the second dose (T1), the day the third dose was administered (T2), and 3 months after the third dose (T3).

**Results:**

In PLWH: i) NA against all variants was higher in SIV compared to SV at T2 and was increased at T3; ii) switched-memory plasmablasts were augmented in SIV alone at T2 and T3; iii) a SARS-CoV-2 specific T cell memory was generated; iv) IFN-γ-secreting CD4+ and CD8+ T lymphocytes were boosted at T3 mainly in SV. CMI magnitude was reduced in PLWH compared to HC. Notably, after the third dose of vaccine viremia was unmodified, but CD4 T cell counts were reduced>20% in 3/29 PHLW.

**Conclusion:**

A third dose of BNT162b2 vaccine induces strong humoral and CMI responses in young ART-treated PLWH independently from a previous SARS-CoV-2 natural infection. The lower magnitude of CMI responses should be considered when planning mRNA vaccine booster doses in PLWH.

## Introduction

Incredibly successful efforts by the scientific community at-large led to the development of safe and effective vaccines that contained the spread of SARS-CoV-2. To date, available data indicate that the BNT162b2 messenger RNA (mRNA) vaccine, the most effective of such vaccines, massively prevents severe COVID-19 and death in the general population (1, 2). Studies on primary vaccination indicate that the BNT162b2 vaccine induces robust and protective SARS-CoV-2-specific humoral and cell-mediated responses in healthy adults (3). A limited number of short-term follow-up studies reported that the BNT162b2 vaccine is well tolerated and is immunogenic in antiretroviral therapy (ART)-treated people leaving with HIV (PLWH) (4) as well, and very scarce results are available in HIV- vertically infected young PLWH (5).

SARS-CoV-2-specific immune responses wane within months from the second dose (6, 7), and the emergence of new variants of concern (VoCs) poses further alarm, as these variants are characterized by increased transmissibility, and are likely to be more prone to immune evasion. The B.1.1.529 (Omicron) variant, in particular, is more infectious (8), and is characterized by a reduced susceptibility to vaccine-induced neutralization and an increased risk of re-infection (7, 9). Thus, even if several studies reported a significant rise in Omicron neutralization after the administration of a third vaccine dose in the general population, such effect was shown to be only partially protective (10–12). Data on the efficacy of a third booster dose of SARS-CoV-2 vaccine in PLWH, and in particular in young, HIV-vertically infected PLWH, are currently limited (13). This is an urgent matter, as many clues suggest that immunization in PLWH might result in suboptimal immune response, reduced durability, and diminished clinical efficacy (14–17).

Another major issue concerns the immunity conferred by past SARS-CoV-2 infection. Results showed that immunity conferred by natural infection is lower against the omicron BA.1 variant and declines rapidly over time. On the other hand, immunity conferred by the combination of previous infection and vaccination, the so called “hybrid immunity”, offers a greater broad-spectrum protection (18, 19), elicits higher levels of neutralizing antibodies (20), and provides more effective protection against infection (21, 22) than immunity conferred by vaccination or infection alone. Whether a hybrid immunity may elicit these same protective effects in our particular population of patients (ART- treated and vertically infected PLWH) remains to be elucidated.

Based on these premises, we performed a longitudinal observational study to evaluate the development of humoral and cellular immune responses induced by three doses of the Pfizer BioNTech BNT162b2 vaccine in a cohort of ART-treated, HIV-vertically-infected, young PLWH patients. We focused our analyses on the major anti-viral immunological mechanisms and on immune memory triggered by vaccines or viral infections. Enrolled individuals were stratified according to the presence/absence of previous SARS-CoV-2 infection. The primary end-point of the study was to verify the nature of SARS-CoV-2 specific immunity triggered by a third (booster) vaccine dose in PLWH, comparing such effect to that elicited by three BNT162b2 doses in a cohort of age-and-sex matched healthy controls.

## Materials and methods

### Enrollment and study design

We performed an observational study between April 2021 and April 2022; 96 ART-treated HIV-vertically infected young patients are followed at the Unit of Pediatric Infectious Diseases, Luigi Sacco Hospital, University of Milan, Milan, Italy. We collected biological samples at different time points from 29 of them who completed the vaccination schedule (first, second and third dose) with the Pfizer BioNTech BNT162b2 vaccine. Results were compared with those obtained in 30 HIV-negative BNT162b2 -vaccinated age-and-sex-matched volunteers (Healthy Controls, HC).

**Figure 1:**
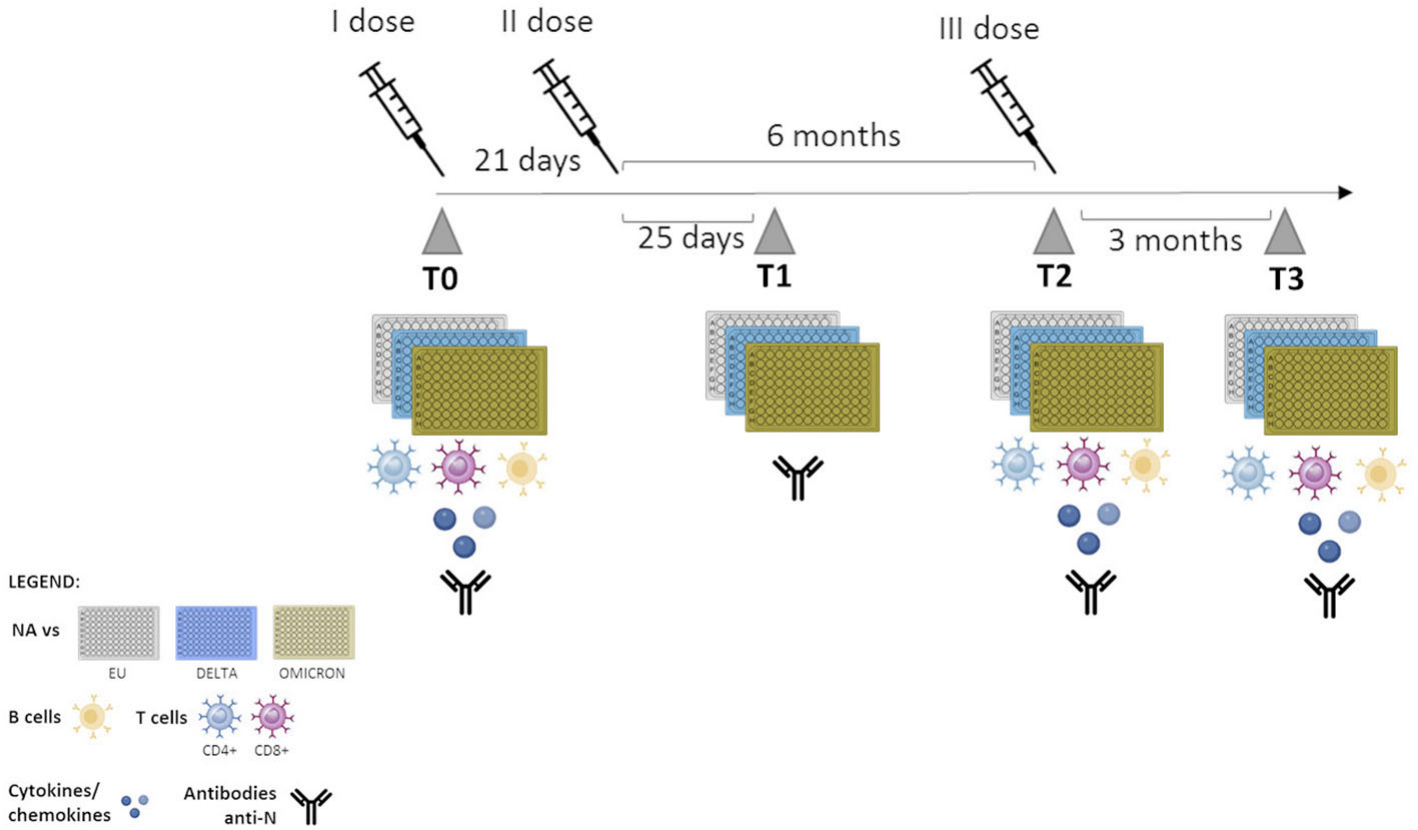
Timeline of sample collection. T0: one day before vaccination; T1: 25 days after the second dose; T2: 6 months after second dose- administration of the third dose, T3: 3 months after the third dose.

Immunological analyses were performed over a 12 month’s period, with the baseline corresponding to T0 (prior to vaccination). After T0, immune responses were evaluated at the following time points: T1 = 25 days after the second dose; T2 = 6 months after second dose and on the day of the third dose was administered; T3 = 3 months after the booster dose) (**Figure 1**). HIV-RNA viral load (VL) detection and CD4+ T cell count were performed in all PLWH at the time of enrollment and after the administration of the third vaccine dose.

PLWH and HC were divided into two sub-groups, based on the detection or lack thereof of serum anti-SARS-CoV-2-Nucleocapsid-specific IgG, as previously described (23) or on a positive result with an antigenic test or RT-PCR test performed on a nasopharyngeal exudate sample: 1) SARS-CoV-2-vaccinated individuals with a previous history of SARS-CoV-2 infection (SIV); and 2) SARS-CoV-2-vaccinated individuals with no history of previous SARS-CoV-2 natural infection (SV).

All SIV subjects enrolled in the study experienced mild symptoms from COVID-19 and didn’t require hospitalization at the time of infection.

This prospective study was approved by the Ethical Committees of Sacco Hospital (protocol number 0034645, approved on 08/11/2020). Research was performed in accordance with the relevant regulations, and informed consent was obtained from all the participants’ parents or their legal guardians. All participants, including healthy volunteers, gave their written informed consent following the Helsinki Declaration.

### SARS-CoV-2 specific antibodies

Serum at T0 was tested for anti-SARS-CoV-2-specific IgG using the semi-quantitative Anti-SARS-CoV-2 ELISA (Euroimmun, Löbeck, Germany) test, according to manufacturer’s instructions. Specifically, 10 µl of each serum was diluted 1:101 in Sample Buffer, and 100 µl of diluted serum were incubated into individual microplate wells coated with an S1 domain of the spike protein of SARS-CoV-2 expressed recombinantly in the human cell line HEK 293. Results were assessed semi-quantitatively by a ratio (Optical Density sample/Optical Density calibrator) and interpreted as follows: <0.8 negative, ≥0.8 to <1.1 borderline, ≥1.1 positive.

To exclude a possible ongoing asymptomatic or symptomatic infection, plasma samples were heat-inactivated for 30 minutes at 56°C and analyzed using an anti-SARS-CoV-2 Nucleocapsid ELISA kit (Immunodiagnostics, Hong Kong).

### Viral strains and cell lines

SARS-CoV-2, including the lineage B.1 (EU), the Delta (lineage B.1.617.2) and Omicron (lineage B.1.1.529) variants were isolated from positive nasopharyngeal swabs. All the strains were characterized by means of whole genome sequencing and the sequences were submitted to GISAID. The virus was expanded in Vero E6 cells (CRL-1586™, African green monkey kidney epithelial cells, ATCC®, Manassas, VA, USA) and viral titers were determined by Median Tissue Culture Infectious Dose (TCID_50_) endpoint dilution as previously described (24, 25). All the assays with SARS-CoV-2 were performed in a BSL3 facility.

### Sample processing and cell culture

Blood samples, collected in a BD Vacutainer Plus plastic serum tube, were processed to separate plasma and peripheral blood mononuclear cells (PBMCs), as previously described (26). Plasma was obtained by centrifugation of whole blood at 400g for 10 min and storage at −20°C until use. Plasma was then employed for neutralization assay, while PBMCs were resuspended at the concentration of 1,5×10^6^ cells/150μl in 10% FBS RPMI 1640 medium (Euroclone, Milan, Italy) and subsequently stimulated with anti-CD28 (1 μg/mL) and 180 µg/mL of SARS-CoV-2 spike (S) recombinant peptides, a mixture of 181 17-/13-mers peptides spanning the full length of the protein (NR-52402, BEI Resources, NIAID, NIH). Unstimulated PBMCs were cultured as negative controls. Cells were harvested 18 hours post-stimulation for flow cytometric analyses.

### SARS-CoV-2 neutralization assay

Plasma samples were thawed at room temperature and heat-inactivated for 30 minutes at 56°C. Neutralization activity (NA) against SARS-CoV-2 B.1 (EU), Delta (lineage B.1.617.2) and Omicron (lineage BA.1) variants was performed by virus neutralization assay (vNTA) at four different time points: T0, T1, T2 and T3. Briefly, two‐fold serial dilutions of plasma samples, starting from 1:10, were mixed with an equal volume of tissue culture infecting dose 50 (TCID50) of SARS-CoV-2 EU, Delta or Omicron variants. All dilutions were made in DMEM High Glucose (Euroclone, Milan, Italy) with the addition of 2 mM L-Glutamine, 100 U/ml of penicillin and streptomycin (Life Technologies), and 2% fetal bovine serum (FBS) (Euroclone, Milan, Italy). The plasma‐virus mixture was incubated for 2 hours at 37°C in a humidified atmosphere with 5% CO2. After incubation, the mixture at each dilution was transferred in duplicate to a 96-well microplate, pre-seeded with 2x10^4^ VeroE6 cells (CRL-1586™, African green monkey kidney epithelial cells, ATCC^®^, Manassas, VA, USA). The plates were incubated for 72 hours at 37°C and 5% CO2. At the end of incubation, VeroE6 cells were fixed with 4% paraformaldehyde (PFA) 37% m/v (Merck KGaA, Darstadt, Germany) for 20 minutes and then stained with 0.1% m/v crystal violet solution (Merck KGaA, 64271 Damstadt, Germany). Microtiter plates were then thoroughly washed with PBS (Euroclone, Milan, Italy). Wells were inspected to evaluate the degree of cytopathic effects (CPE) compared to the virus control. Neutralizing titer corresponds to the maximum dilution with the reduction of 90% of CPE. A positive titer was equal to or greater than the dilution of 1:10, and dilution are expressed as absolute values in the graphs. Every test included plasma control (1:10 dilution), cell control (VeroE6 cells alone) and viral control (TCID50, three-fold series dilution).

### Flow cytometry

A complete evaluation of SARS-CoV-2-specific peripheral B and T cell subsets was performed in a subgroup of 23 patients at baseline (T0), T2 and T3. Results at T2 and T3 were compared to those obtained from 14 HCs. SARS-CoV-2–specific B and T cells were identified as previously described (27, 28). Briefly, frozen PBMCs were thawed and allowed to rest overnight at 37°C in a humidified atmosphere with 5% CO2 in a complete medium characterized by RPMI 1640 (Euroclone, Milan, Italy), supplemented with 10% FBS (Euroclone, Milan, Italy), 2 mM L-glutamine and 100 U/ml penicillin and streptomycin (Life Technologies). PBMCs were then resuspended at the concentration of 1,5x10^6^ cells/mL in 10% FBS RPMI 1640 medium (Euroclone, Milan, Italy) and subsequently stimulated with anti-CD28 (1 μg/mL) and 180 µg/mL of SARS-CoV-2 spike (S) recombinant peptides, a mixture of 181 17-/13-mers peptides spanning the full length of the protein (NR-52402, BEI Resources, NIAID, NIH). Unstimulated PBMCs were cultured as a control. During the last 6 hours of stimulation, 1μg/ml of Brefeldin A (Sigma-Aldrich) was added to block protein secretion. After 18 hours, PBMCs were harvested and incubated with monoclonal antibodies to detect surface antigens for 15 minutes at room temperature, protected by light. Then, cells were washed with PBS (Euroclone, Italy) and fixed in 1% paraformaldehyde (PFA, Sigma-Aldrich, MO, USA). Otherwise, cells were permeabilized (eBioscience™ Foxp3/Transcription Factor Staining Buffer Set) and stained with antibodies to detect intracellular IFN-γ for 30 minutes at room temperature and then fixed with 1% PFA. The following anti-human antibodies were selected: CD45 Krome Orange (Beckman Coulter), CD4 PC5.5 (Invitrogen), CD8 PC7 (Beckman Coulter), CD45RA FITC (Beckman Coulter), CCR7 PE (R&D Systems), CD19 FITC (Beckman Coulter), CD20 PC7 (Beckman Coulter), CD24 ECD (Beckman Coulter), CD38 PC5.5 (Beckman Coulter), IgD APC (Beckman Coulter), CD21 APC Alexa Fluor 750 (Invitrogen), CD25 PE (Becton Dickinson, BD), CD45RO FITC (Beckman Coulter), CD107a Pacific Blue (Beckman Coulter), CD38 SNv605 (Beckman Coulter) and IFNγ APC (Biolegend). Samples acquisition was performed on a CytoFLEX™ flow cytometer system equipped with CytExpert software (Beckman Coulter), and data were analyzed using Kaluza software, version 2.1.1. (Beckman Coulter).

### Multiplex cytokine analyses

A 17-cytokine multiplex assay was performed on culture supernatants from patients, using a multiplexed Luminex magnetic bead immunoassay (Bio-Rad, CA, USA) according to the manufacturer’s instructions.

### Statistical analysis

The Student’s t-test was done when appropriate for statistical analysis to compare continuous and categorical variables. One-way ANOVA or Two-way ANOVA were applied for non-parametric multiple comparisons. Spearman correlation was used to examine the association between variables/features. A p-value < 0.05 was chosen as the cutoff for significance. Data were analyzed with GraphPad Prism 9.

## Results

### Clinical characteristics of study population

Ninety-six vertically-infected PLWH are currently followed at our unit (23); twenty-nine of them were enrolled in the study (mean age 27,9 years; range 17- 39 years) and received three doses of the BNT162b2 mRNA-vaccine (**Table 1**). Sixteen of these individuals reported no history of SARS-Co-CV-2 infection prior to vaccination (SARS-CoV-2 vaccinated -SV-), whereas 13 of them had been SARS-CoV-2-infected before administration of the first vaccine dose (SARS-CoV-2 infected and vaccinated -SIV-). We excluded ongoing asymptomatic or symptomatic infections during the study period (the enrolled subjects resulted negative for anti-SARS-CoV-2 Nucleocapsid antibodies).

**Table 1:** Clinical characteristics of the study population.

	PLWH (29)		HC (30)	
	SV (16)	SIV (13)	SV (15)	SIV (15)
Age (years, mean ± SD)	29.6 ± 7.6	25.5 ± 6	26.6 ± 8.4	28 ± 11
Females/Males	10/6	8/5	10/5	9/6
Absolute CD4+ T cell count (cells/mm^3^ ± SD) T0 T3	460 ± 312.9 767.3 ± 333	891 ± 367.2 820.5 ± 259.3	N/A	N/A
CD4+ T cell percentage (% ± SD) T0 T3	40.6 ± 5.9 32.7 ± 10.4	39.2 ± 7 38.2 ± 5.6	N/A	N/A
CD4+ T cell count * < 350 mm^3^ (detectable VL) * T0 T3 350 - 500 mm^3^ (detectable VL) * T0 T3 > 500 mm^3^ (detectable VL) * T0 T3	3 (2) 2 (1) 2 (0) 2 (0) 11 (1) 12 (1)	0 (0) 1 (0) 2 (0) 1 (0) 11 (2) 11 (0)	N/A	N/A
Individuals with HIV-RNA <20 cp/ml T0 T3	13 14	11 13	N/A	N/A
Type of Antiretroviral Therapy* PI INI NNRTI	4 10 2	4 7 2	N/A	N/A

*number of individuals.

PI, Protease inhibitor; INI, Integrase inhibitors; NNRTI, Non-nucleoside reverse transcriptase inhibitors.

All PLWH were receiving ART and 24/29 were virologically suppressed at the time of immunization; viremia became undetectable in three of these 5 individuals during the 12 follow up months (SV=1; SIV=2), and the three doses of vaccine did not provoke an increase in viremia in any of the PLWH analyzed. At the time of enrollment, CD4 cell count was > 500 cells/mm^3^ in 22 individuals (three of them with detectable VL), < 350 cells/mm^3^ in three patients (two of three with detectable VL), and between 350 and 500 cells/mm^3^ in the remaining four patients. After the administration of the third vaccine dose, CD4 count were < 350 cells/mm^3^ in three individuals (one of them with detectable VL), and between 350 and 500 cells/mm^3^ in three more PLWH. One of the patients with CD4 cell count > 500 cells/mm^3^ reported detectable HIV VL.

Contrary to the lack of any evident effect of vaccination on viremia, after the third dose of vaccine a decline >20% in absolute CD4 T lymphocytes count was detected in 3/29 (10%) PLWH (patients #45, #21, #54, in **Supplementary Table 1**); in two of these patients CD4 T lymphocyte percentage declined as well (patients #45 and #21 in **Supplementary Table 1**).

Among the 30 enrolled healthy controls (mean age 27.2 years; range 15- 49 years) 15 were SV and 15 were SIV. No comorbidities were reported.

### SARS-CoV-2-specific neutralizing activity in SV and SIV

**Figure 2:**
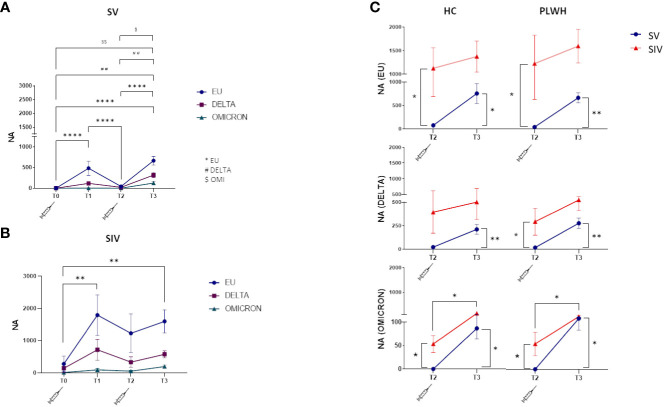
Neutralizing activity against EU, Delta and Omicron variants. Plasma neutralizing activity (NA) over time against EU (blue line), Delta (purple line) and Omicron (green line) in SV **(A)** and SIV **(B)** PLWH; **(C)** Comparison in plasma neutralizing activity (NA) between SV (blue line) and SIV (red line) at T2 and T3 against the three variants in HC (left panels) and PLWH (right panels). Mean values ± SEM are reported. Significance was set at P < 0.05 (Two-way ANOVA test, p values adjusted for multiple comparisons). *p<0.05, **p<0.01, ***p < 0.001, ****p < 0.0001; ^##^p<0.01, ^###^p < 0.001; ^$^p < 0.05, ^$$^p<0.01.

NA against all three SARS-CoV-2 variants tested (EU, Delta, Omicron) was detected at T1, greatly declined at T2, and was boosted at T3 by the third dose of vaccine in all individuals. NA against all variants was more potent in SIV compared to SV PLWH and HC individuals, while no significant overall differences could be observed between PLWH and HC (**Figures 2A, B**; **Supplementary Figure S1**).

In details, NA was greatly diminished in SV six months after the two doses of vaccine (T2), but this trend was reversed at T3. NA was increased against both the EU (T3 vsT2: p<0.0001; T3 vs T0: p<0.0001) and the DELTA variants (T3 vs T2: p<0.01; T3 vs T0: p<0.001) after the third booster vaccine dose (**Figure 2A**). NA was decreased is SIV as well at T2 compared to T1, and was greatly increased at T3 (EU variant, T3 vs T0: p<0.01) (**Figure 2B**).

Notably, and confirming the less than perfect ability of the vaccine to stimulate optimal immune responses against the Omicron variant, NA levels were significantly lower against this variant in all the groups analyzed (vs. European variant p<0.0001 in SV and p<0.05 in SIV) (**Supplementary Figure S2**).

The weaker effect toward Omicron notwithstanding, NA was significantly higher at T2 in PLWH and HC SIV compared to PLWH and HC SV for all the three variants (**Figure 2C**), and the third dose elicited significantly higher NA at T3 compared to T2 in all individuals. Thus, at T3, NA against all the three variants was increased in all individuals (**Figure 2C**), confirming the important booster effect of a third BNT162b2 vaccine dose.

Interestingly, NA could not be detected in one patient with < 350 cells/mm^3^ CD4 and detectable VL after 2 vaccine doses (T2) (patient #22 in **Supplementary Table 1**), but was strongly elicited against all three viral variants by the third vaccine dose.

### B-lymphocytes phenotypic profile

**Figure 3:**
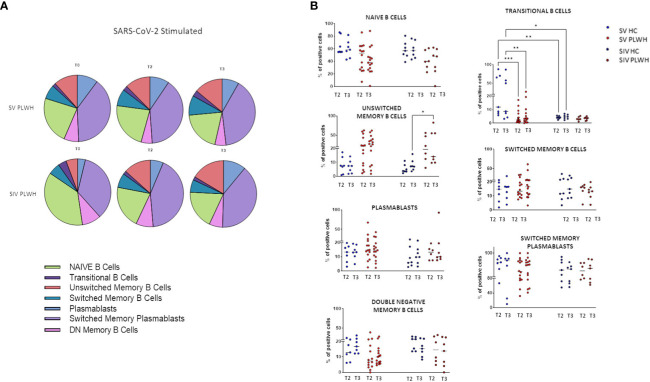
B-cell phenotypic profile. **(A)** Pie charts representing the different B cell subsets at T0, T2 and T3 in SV (n=15) and SIV (n=8) PLWH upon SARS-CoV-2 stimulation; mean values are reported. **(B)** Longitudinal analysis of the frequency (% of positive cells) of B cell subsets at T2 and T3 in SV and SIV HC (blue dots) and PLWH (red dots); interleaved scatterplot graphs depicting median and p-values are reported. Significance was set at P < 0.05 (Two-way ANOVA test, p values adjusted for multiple comparisons). *p<0.05, **p<0.01, ***p < 0.001.

To better understand the quality of the humoral response induced by the BNT162b2 vaccine, we assessed immunization-induced changes in the frequency of SARS-CoV-2-specific B lymphocyte subsets in all the individuals enrolled in the protocol by flow cytometry. As compared to T0, significantly higher frequencies of SARS-CoV-2-specific switched memory plasma blasts were observed in SIV PLWH over time (**Figure 3A**; **Supplementary Table 2**).

Both in HC and PLWH SIV individuals, the potency of Omicron variant-specific NA was positively correlated to the percentage of switched memory plasma blasts at T3 (Spearman R=0.8571, p=0.0238) (**Supplementary Figure S3**). Of note, no increment in exhausted double negative B memory cells (29) was observed during the follow up in any of the analyzed groups of individuals.

After vaccination significant higher percentages of transitional memory B lymphocytes were observed at all time-points in SV HC compared to SV PLWH (T2 p<0.001; T3 p<0.01) and to SIV HC (T2 p<0.0001; T3 p<0.05) (**Figure 3B**). Conversely, unswitched memory B lymphocytes were significantly expanded at T3 in SV PLWH (vs SIV HC: p<0.05).

### SARS-CoV-2-specific cell-mediated immune responses: T lymphocyte phenotypes

**Figure 4:**
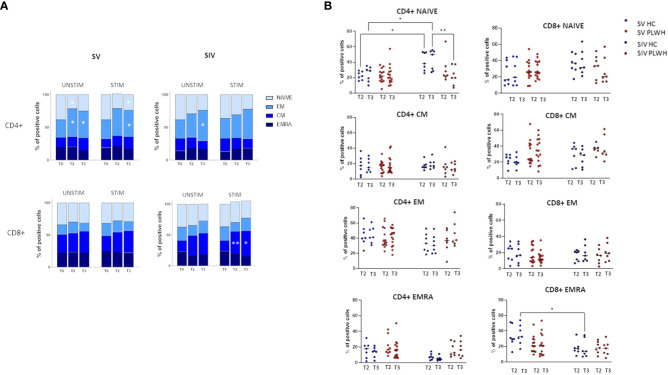
T cell subsets. **(A)** CD4+ (upper panels) and CD8+ (lower panels) T cells memory subsets in SV (left panels) and SIV (right panels) PLWH in the absence (UNSTIM) or presence (STIM) of a 24 hours *in vitro* stimulation with SARS-CoV-2 peptides throughout the time; mean and p values to baseline are reported. **(B)** Longitudinal analysis of the frequency (% of positive cells) of T-cell subsets at T2 and T3 in SV and SIV HC (blue dots) and PLWH (red dots); interleaved scatterplot graphs depicting median and p-values within each group are reported. Significance was set at P < 0.05 (Two-way ANOVA test, p values adjusted for multiple comparisons). *p<0.05, **p<0.01.

T lymphocyte memory subpopulations were evaluated in both SV and SIV PLWH and HC at T0, T2 and T3 by flow cytometry. In SV PLWH, a significant increase in SARS-CoV-2-specific CD4+ effector memory (CD4+CCR7-CD45RA-) (EM) and a significant decline in CD4+ naive (CD4+CCR7+CD45RA+) T lymphocytes was detected at T3 compared to T0 (p<0.05) (**Figure 4A**).

SARS-CoV-2 specific CD8+ central memory (CD8+CCR7+CD45RA-) (CM) T lymphocytes were significantly increased in SIV PLWH both at T2 (p<0.01) and at T3 (p<0.05) compared to T0 (**Figure 4A**). No significant differences were observed in CD4+ and CD8+ T lymphocytes subpopulations when PLWH were compared to HC after the third BNT162b2 vaccine dose, even if CD4+ naïve T lymphocytes were significantly reduced at T3 in SIV PLWH compared to SIV HC (p<0.01) (**Figure 4B**).

### SARS-CoV-2-specific cell-mediated immune responses: T lymphocyte function

To better characterize T lymphocytes-mediated SARS-CoV-2 BNT162b2 immunogenicity, we also assessed SARS-CoV-2–specific IFN-γ-secreting CD4+ and CD8+ T lymphocytes by flow cytometry.

**Figure 5:**
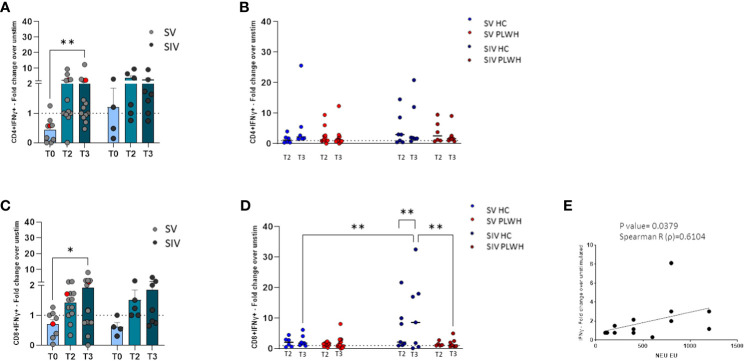
T cell-mediated immune response. SARS-CoV-2 specific IFN-γ+ secreting CD4+ **(A)** and CD8+ **(C)** T cells represented as fold change over unstimulated in SV (light grey dots) and SIV (dark grey dots) PLWH, and patient #45 (red dots), throughout the time; mean values ± SEM are reported. Longitudinal analysis of the SARS-CoV-2 specific IFN-γ+ secreting CD4+ **(B)** and CD8+ **(D)** T cells represented as fold change over unstimulated at T2 and T3 in SV and SIV HC (blue dots) and PLWH (red dots); interleaved scatterplot graphs depicting median and p-values are reported. **(E)** XY scatter plot that shows the relationship between neutralizing activity against EU variant and SARS-CoV-2 specific IFN-γ+ secreting CD8+ cells in SV PLWH at T3. The scatter plot reports the regression line (black), the Spearman R (ρ) value and the exact two-tailed P-value. Significance was set at P < 0.05. *p<0.05, **p<0.01.

A statistically significant increase of IFN-γ-secreting CD4+ T lymphocytes (p<0.01, **Figure 5A**) was detected in SV PLWH at T3 compared to T0, and a similar trend was observed in SIV PLWH (**Figure 5A**). No differences were observed between SV and SIV PLWH (**Figure 5A**). IFN-γ-secreting CD4+ T lymphocytes at T2 and T3 were increased in HC compared to PLWH SIV and SV; these differences approached but did not reach statistical significance (**Figure 5B**).

Similarly, an increase of IFN-γ-secreting CD8+ T lymphocytes was observed at T3 compared to T0 in PLWH, reaching statistical significance in the SV group (p<0.05, **Figure 5C**). SARS-CoV-2-specific IFN-γ-secreting CD8+ T lymphocytes in SIV were significantly increased at T3 compared to T2 (p<0.01) (**Figure 5D**) and, at T3, were significantly higher in SIV HC compared to SIV PLWH (p<0.01) and SV HC (p<0.01) (**Figure 5D**). Finally, in SV PLWH, the percentage of SARS-CoV-2 specific IFN-γ+CD8+ T lymphocytes correlated with NA against the EU variant at T3 (p<0.05; Spearman R=0.6104) (**Figure 5E**).

Notably, potent vaccine-elicited cell-mediated immune responses could be detected both at T2 and T3 in the most immunocompromised PLWH individual, even in patient #45 (**Supplementary Table 1**), who had a CD4 count barely >200 mm^3^ and a viremia >800 cp/ml (red dots in **Figures 5A, C**).

Finally, CD8+/CD107+ T lymphocytes and activated CD4+ (CD4+/CD25+) and CD8+ (CD8+/CD38+) T lymphocytes were not modified by the three vaccine doses in either HC or PLWH (**Supplementary Figure S4**).

### Cytokine profiles in PLWH and HC

**Figure 6:**
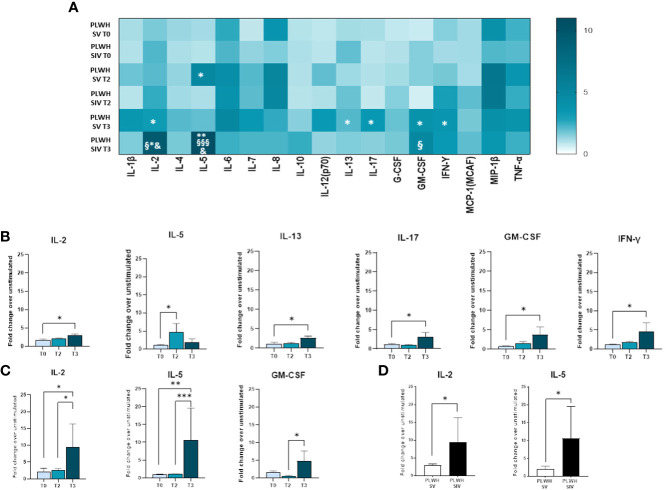
Cytokine and chemokine profiles in SV and SIV PLWH. SARS-CoV-2 specific cytokines and chemokines production overtime from supernatants of PBMCs isolated from PLWH SV and SIV represented as fold change over unstimulated condition and shown as a color scale from white to dark green (Heatmap) **(A)**. Mean values are reported. Significance was set at P < 0.05 (Two-way ANOVA test, p values adjusted for multiple comparisons). *p<0.05 vs T0, **p<0.01 vs T0; § p<0.05 vs T2; §§§ p<0.001 vs T2; & p<0.05 vs PLWH SV T3. Significant SARS-CoV-2 specific cytokines and chemokines represented as fold change over unstimulated condition in SV **(B)** and SIV **(C)** PLWH throughout the time. **(D)** Significant SARS-CoV-2 specific cytokines represented as fold change over unstimulated in SV (white bars) and SIV (black bars) PLWH at T3. Mean values ± SEM are reported. Significance was set at P < 0.05 (One-way ANOVA test, p values adjusted for multiple comparisons). *p<0.05, **p<0.01; ***p < 0.001.

Modulation of cytokine and chemokine profiles induced by three doses of the BNT162b2 vaccine was analyzed in supernatants of SARS-CoV-2-stimulated PBMCs from PLWH and HC by Multiplex assay. In SV PLWH, the third vaccine dose (T3) induced a significant increase in the production of a number of cytokines compared to the baseline value (T0). Thus, at T3 IL-2, IL-5, IL-13, IL-17, GM-CSF, and IFN-γ (p<0.05) were all significantly increased in this PLWH group (**Figures 6A, B**).

A robust and significant increase in the release of several cytokines and chemokines was observed in SIV PLWH as well (IL-2: p<0.05 T3 vs T0, p<0.05 T3 vs T2; IL-5: p<0.01 T3 vs T0, p<0.001 T3 vs T2; GM-CSF: p<0.05 T3 vs T2) (**Figures 6A, C**).

**Figure 7:**
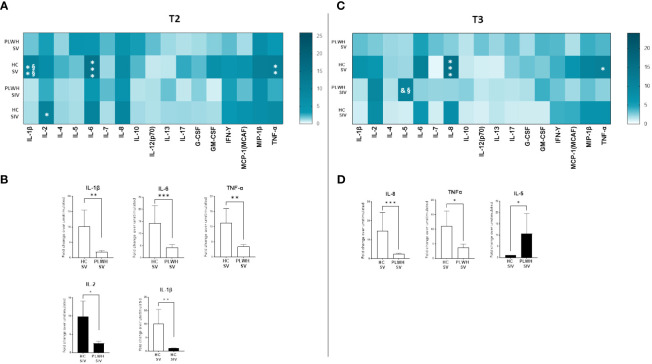
Cytokine and chemokine profiles in PLWH and HC. SARS-CoV-2 specific cytokines and chemokines production from supernatants of PBMCs isolated from SV and SIV PLWH and HC represented as fold change over unstimulated condition and shown as a color scale from white to dark green (Heatmap) at T2 **(A)** and T3 **(C)**. Mean values are reported. Significance was set at P < 0.05 (Two-way ANOVA test, p values adjusted for multiple comparisons). *p<0.05 vs PLWH, **p<0.01 vs PLWH; ***p < 0.001 vs PLWH; § p<0.05 vs HC SIV; §§ p<0.01 vs HC SIV; & p<0.05 vs PLWH SV. Significant SARS-CoV-2 specific cytokines and chemokines represented as fold change over unstimulated condition in SV (white bars) and SIV (black bars) PLWH or HC at T2 **(B)** and T3 **(D)**. Mean values ± SEM are reported. Significance was set at P < 0.05 (Student’s t-test). *p<0.05, **p<0.01; ***p < 0.001.

SARS-CoV-2-stimulated PBMCs of SIV were characterized by a higher production of IL-2 (p<0.05) and IL-5 (p<0.05) compared to SV at T3 (**Figure 6D**). Interestingly, distinctive secretory profiles were observed in PLWH compared to HCs. In particular, at T2 SARS-CoV-2-stimulated PBMCs of SV HCs produced higher amount of IL-1β (p<0.01), IL-6 (p<0.001), and TNF-α (p<0.01) compared to SV PLWH, and IL-2 production (p<0.05) was significantly increased in HC SIV compared to PLWH SIV. IL-1β production was also significantly higher in HC SV (p<0.01) compared to HC SIV (**Figure 7B**). Finally, IL-8 (p<0.01) and TNF-α (p<0.05) production was significantly increased in SV compared to SV PLWH at T3, whereas IL-5 (p<0.05) was significantly increased in SIV PLWH compared to SIV HC at the same time point (**Figures 7A–D**).

### Correlations between cellular and humoral immune parameters

**Figure 8:**
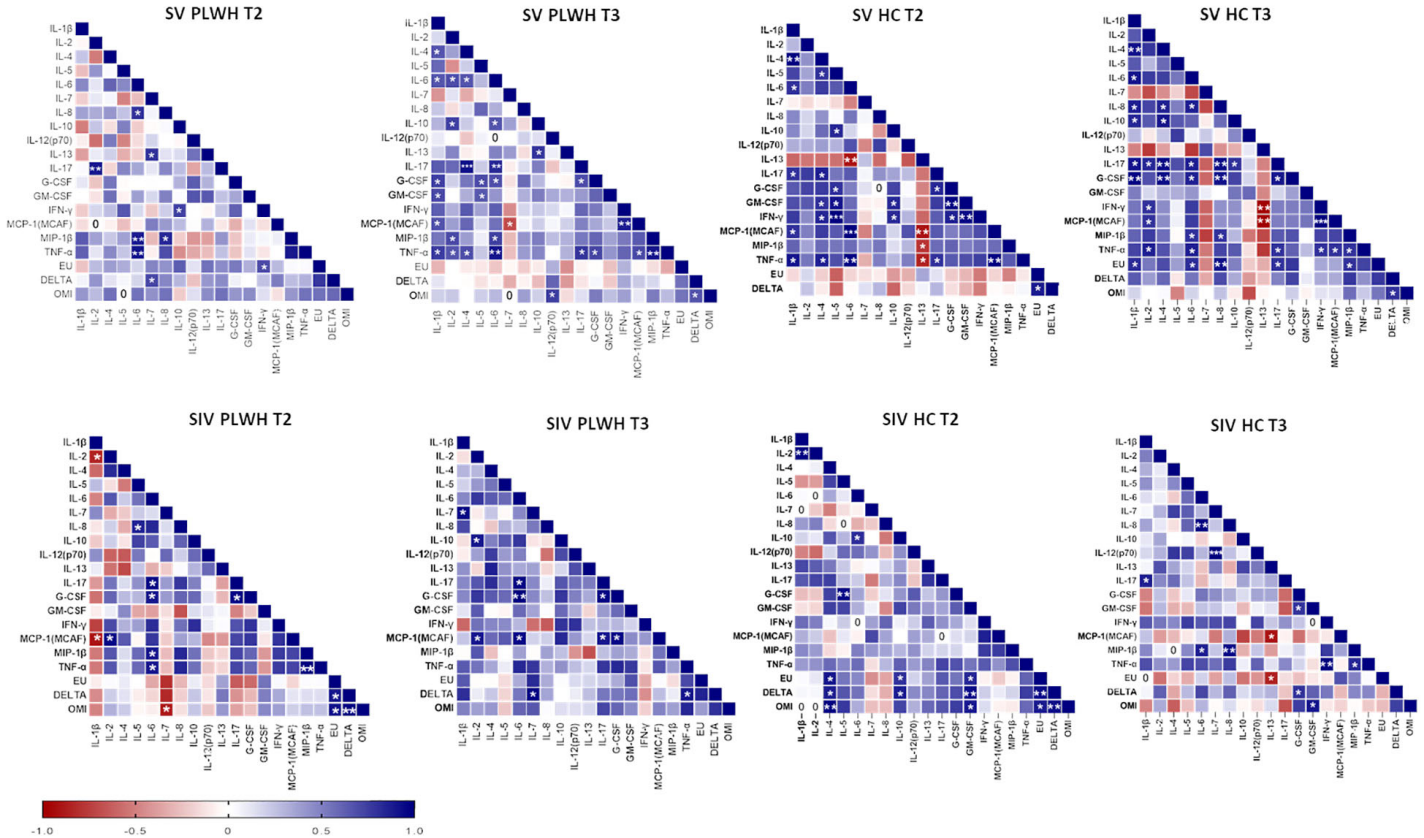
Correlograms of the humoral and cellular immune parameters. Spearman R (ρ) values in SV (upper panels) and SIV (lower panels) PLWH and HC at T2 and T3 are shown from red (−1.0) to blue (1.0); color intensity is proportional to correlation coefficients R. Spearman rank two-tailed P-value was indicated by *p < 0.05, **p < 0.01, and ***p < 0.001.

Several positive correlations among cytokines and NA were found by Spearman correlation analysis (**Figure 8**). Of note, significant positive correlations were observed between NA against the three SARS-CoV-2 variants and IL-4, IL-10 and GM-CSF in SIV HC at T2 (p<0.05 in all cases) and, after the third dose, NA against the DELTA and Omicron variants positively correlated with G-CSF and GM-CSF (p<0.05 in both cases).

Finally, no correlation was found between current or nadir CD4+ T lymphocyte count and NA, IFN-γ-secreting T lymphocytes, or cytokine secretion by PBMCs.

## Discussion

Limited results are available on the efficacy of the third dose of SARS-CoV-2 vaccine in PLWH; even fewer studies have analyzed the efficacy of such vaccines in vertically-infected young PLWH. Data herein indicate that three doses of the BNT162b2 mRNA vaccine are able to elicit robust humoral and cell–mediated immune responses in ART-treated, HIV-vertically-infected young patients. Our results also confirm that SARS-CoV-2 infection before vaccination, resulting in the so-called hybrid immunity, is associated with the development of stronger immune responses compared to vaccination alone, as previously reported in the general population (30, 31). Two important and novel results emerging from our analyses are: 1) while vaccine-induced humoral immune responses are comparable in PLWH and HC, cell-mediated immunity is qualitatively different and, possibly weaker in PLWH; and 2) while vaccination does not reactivate viral replication, it can result in a non-marginal reduction of CD4+ T lymphocytes in a minority of PLWH.

A growing body of evidence suggests that in adult PLWH with normal CD4+ T-lymphocyte counts, both humoral and cell-mediated immune responses elicited by two doses of vaccine are comparable to those observed in healthy individuals (5, 32). The observation that no significant correlation was observed between nadir or actual CD4+ T-lymphocyte counts and vaccine-induced SARS-CoV-2-specific immune responses further reinforces these findings. Nevertheless, controversial data have been reported on the role of a third dose of mRNA vaccine in eliciting neutralizing antibodies and cross-reactive T cell response in PLWH receiving suppressive ART, especially in those showing severe immune dysregulation (33).

Overall, our results show that, albeit vaccine-induced immune responses waned months after the second dose both in PLWH and HC, the third vaccination improves NA to all the considered SARS-CoV-2 variants, including Omicron both in PLWH and HCs. Therefore, potential immune escape in Omicron variant- infected individuals is overcome by the administration of the third vaccine dose, which significantly boosts neutralizing activity against the Omicron strain also in PLWH, consistent with the data available from the general population (7, 11, 34). Interestingly, SV individuals mostly benefit from the third dose, which appears to be crucial in increasing NA. Indeed, NA was only modestly boosted by the third vaccine in those individuals who had been SARS-CoV-2 infected prior to vaccination, suggesting that natural infection-induced NA levels are potent and are only marginally increased by a booster vaccine dose (21, 35).

Recent findings in the general population reported that the third dose confers the same protection level observed in previously infected vaccinated individuals (36, 37), although stimulating a peculiar pattern of B lymphocyte subpopulation differentiation (37). In this regard, it is known that HIV induces multiple phenotypic and functional defects that affect B lymphocytes, altering their profiles and their functions (29, 38–41). A skewing toward a more differentiated memory phenotype together with a reduction in naïve B cells was observed in HIV infection (29, 42, 43). Moreover, increased percentages of double negative memory B lymphocyte subsets were shown to represent a marker of exhaustion in ART non-responder PLWH (29), and ART was observed to largely normalize the distribution of B lymphocyte memory subsets (44). Our results show comparable percentages of mature B lymphocytes, switched memory plasma blasts and double negative memory B lymphocytes between HC and PLWH after the third dose of vaccine. Similarly, in a previous study (45) performed six months after the administration of the second dose, the authors found comparable rates of spike-specific B cells in PLWH and HC, yet with different phenotypes (lower switched memory cell frequencies and higher double negative cells in PLWH compared to HC). Although their cohort of PLWH showed optimal immunological recovery, the atypical phenotypes, apparently opposed to our findings, might be related to the older age (mean age 52 years versus 29.6 years of our cohort) and the time from ART initiation, as our patients are HIV vertically-transmitted and started ART at very young age. Another interesting data is related to the percentage of transitional B lymphocytes. The lower frequency in transitional B-cells observed in SIV compared to SV HC supports a higher B-cell differentiation due to the prior infection. On the other hand, the lower transitional B cells observed in both SV and SIV PLWH could be instead explained by a dysfunctional maturation, albeit limited, as this atypical immunophenotype is not accompanied by increased levels of double negative memory B lymphocytes. Notably, the positive correlation between Omicron-directed NA and the increased switched memory plasma blasts population seen at T3 would suggest that the third dose stimulates a preexisting population of cross-reactive SARS-CoV-2-specific memory B lymphocytes (35, 46).

It is now well established that even if vaccine-induced humoral response substantially declines in time after the second vaccine dose, T-lymphocytes responses persist (47). We show that both SV and SIV PLWH develop a robust SARS-CoV-2 specific T-cell memory overtime, which is already present after the first two doses of vaccine. T-lymphocyte subset distribution in PLWH did not differ from that observed in healthy controls, supporting previous results obtained upon analyzing the effect of primary vaccination in a population of HIV-vertically infected patients (5). Notably, the decrease in neutralization activity observed six months after the second dose was apparently compensated by a more potent cellular immune response, characterized by high level of SARS-CoV-2-specific IFN-γ-secreting T-lymphocytes. Other interferons (*i.e.* type I interferons) were not investigated, but some authors reported contrasting results related to SARS-CoV-2 vaccines (48, 49).

Magnitude of vaccine-induced responses was different when comparing PLWH and HC who were or were nor previously SARS-CoV-2 infected. Two doses of vaccine have been shown to be able to elicit persistent and robust cellular immune memory and greater levels of IFN-γ-producing T lymphocytes in individuals with a history of natural SARS-CoV-2 infection (46, 50, 51). Here, higher percentages of IFN-γ-secreting CD8+ T lymphocytes were seen in SIV HC compared to SV HC. Most importantly, the overall magnitude of the vaccine-induced cell mediated immune response was marginally but consistently reduced at all time-points in PLWH compared to HC. This might be the consequence of an incomplete immune reconstitution on ART that could hamper the vaccine effectiveness in PLWH, as previously reported (52). This was confirmed by evaluating the secretome in supernatants. Thus, PBMC of HC produced higher amounts of cytokines and chemokines upon SARS-CoV-2 stimulation, and the overall profile of immune proteins produced by HC and PLWH was qualitatively different. The only cytokine found to be significantly higher in SIV PLWH compared to the HC is IL-5. We can speculate that this result may be related to a prevalent Th2-like response and to a granulocyte involvement - as suggested by the increase in GM-CSF - in PLWH (53–55). The ability of SARS-CoV-2-specific mRNA vaccines to elicit protective immune responses in PLWH notwithstanding, these data indicate the need to design protocols targeting PLWH that take this potential residual immune deficit into consideration.

Transient increases in HIV cell-associated RNA has been reported following vaccination for influenza (56, 57), streptococcus pneumoniae (58), and hepatitis B virus (59). Our results indicate that HIV viremia was not affected by multiple doses of BNT162b2 mRNA vaccine. These data are in agreement with recently published results (5) and, although not every report agrees (60, 61), suggest that this vaccination protocol does not represent a risk in PLWH as far as viremia is concerned. Potentially more worrisome is the observation that noticeable drops in CD4 counts could be detected in a small but not negligible percentage of ART-treated patients. This possible negative effect of SARS-CoV-2 vaccination will need to be analyzed in-dept, as it might be an important red flag raised on the use of repeated doses of mRNA vaccine in PLWH.

This study has some limitations. First, the subgroups of SV and SIV are small, and further studies on larger cohorts should be considered to better delineate immune profile characteristics of these groups. Second, our study covers a period lasting up to three months after the third dose. It would be interesting to extend our study for a longer period in order to have more in-depth information on the persistence of immune memory.

In conclusion, our data indicate that three doses of BNT162b2 mRNA SARS-CoV-2 vaccine in HIV-vertically infected, ART-treated young patients result in the generation of robust SARS-CoV-2-specific humoral and cell-mediated immune responses. The different magnitude and quality of CMI immune responses elicited by vaccination in PLWH might need to be taken into account when designing vaccine protocols in PLWH, in whom CD4 counts will also need to be carefully monitored.

## Data availability statement

The original contributions presented in the study are included in the article/**Supplementary Material**. Further inquiries can be directed to the corresponding author.

## Ethics statement

This prospective study was approved by the Ethical Committees of Sacco Hospital (protocol number 0034645, approved on 08/11/2020). Research was performed in accordance with the relevant regulations, and informed consent was obtained from all the participants’ parents or their legal guardians. All participants, including healthy volunteers, gave their written informed consent following the Helsinki Declaration.

## Author contributions

CV: Data curation, Formal Analysis, Investigation, Methodology, Writing – original draft. MS: Data curation, Investigation, Writing – original draft. EC: Investigation, Writing – original draft. FC: Investigation, Writing – review & editing. MG: Formal Analysis, Investigation, Writing – original draft. CF: Validation, Writing – review & editing. MB: Validation, Writing – review & editing. VR: Investigation, Writing – review & editing. AA: Formal Analysis, Investigation, Writing – review & editing. ET: Formal Analysis, Investigation, Writing – review & editing. GZ: Resources, Writing – review & editing. MC: Conceptualization, Resources, Validation, Writing – original draft, Writing – review & editing. VG: Conceptualization, Supervision, Validation, Writing – original draft, Writing – review & editing. DT: Conceptualization, Supervision, Validation, Writing – original draft, Writing – review & editing.
