# Supplementary figures and images for: Humoral and cell-mediated immune responses in HIV-vertically infected young patients after three doses of the BNT162b2 mRNA SARS-CoV-2 vaccine
Source: Front Immunol. 2024 Jan 4;14:1301766. doi: 10.3389/fimmu.2023.1301766 (PMC10797701; doi:10.3389/fimmu.2023.1301766)

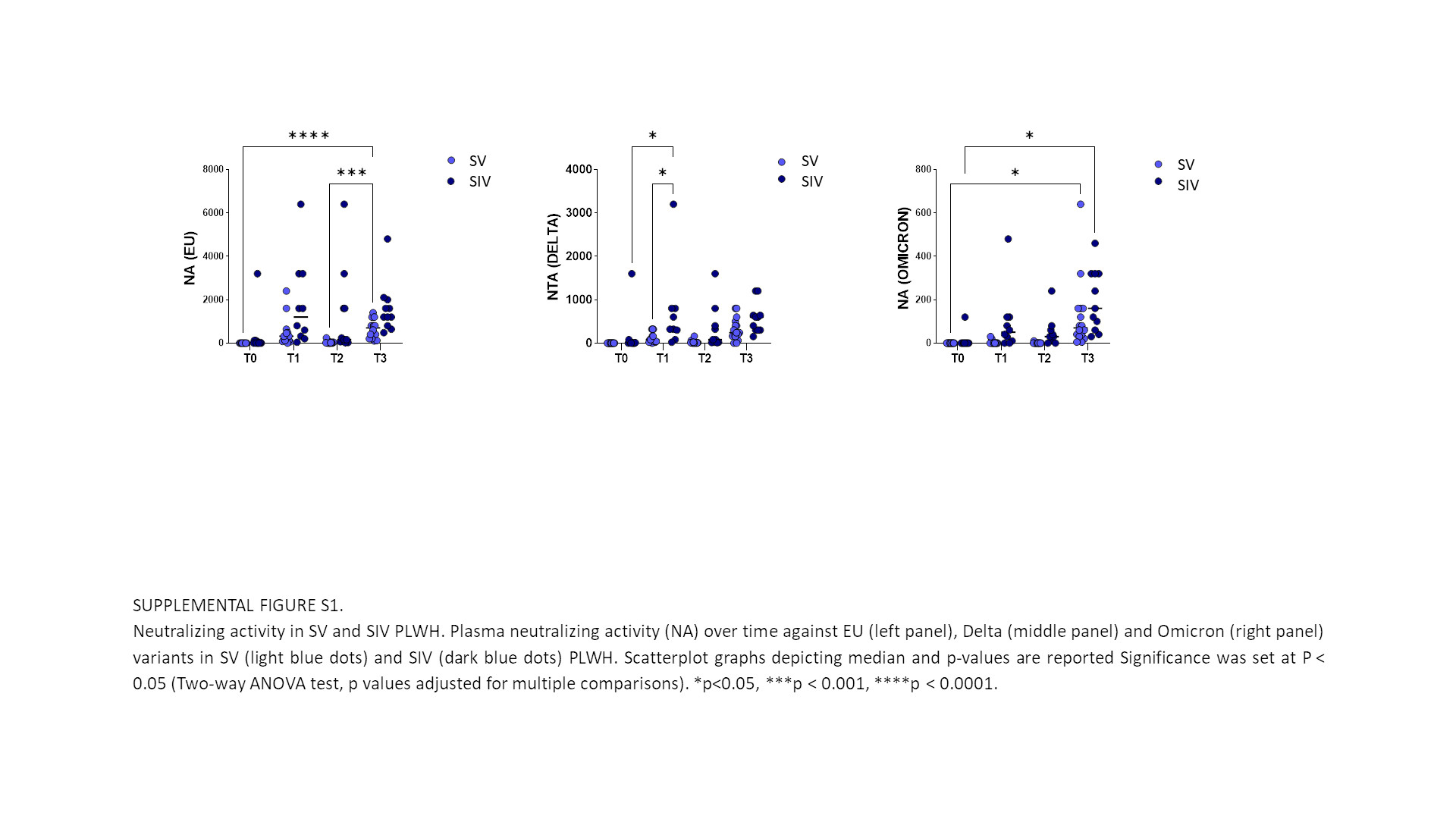

Supplement: Supplementary file 1 [file Image_1.jpg]

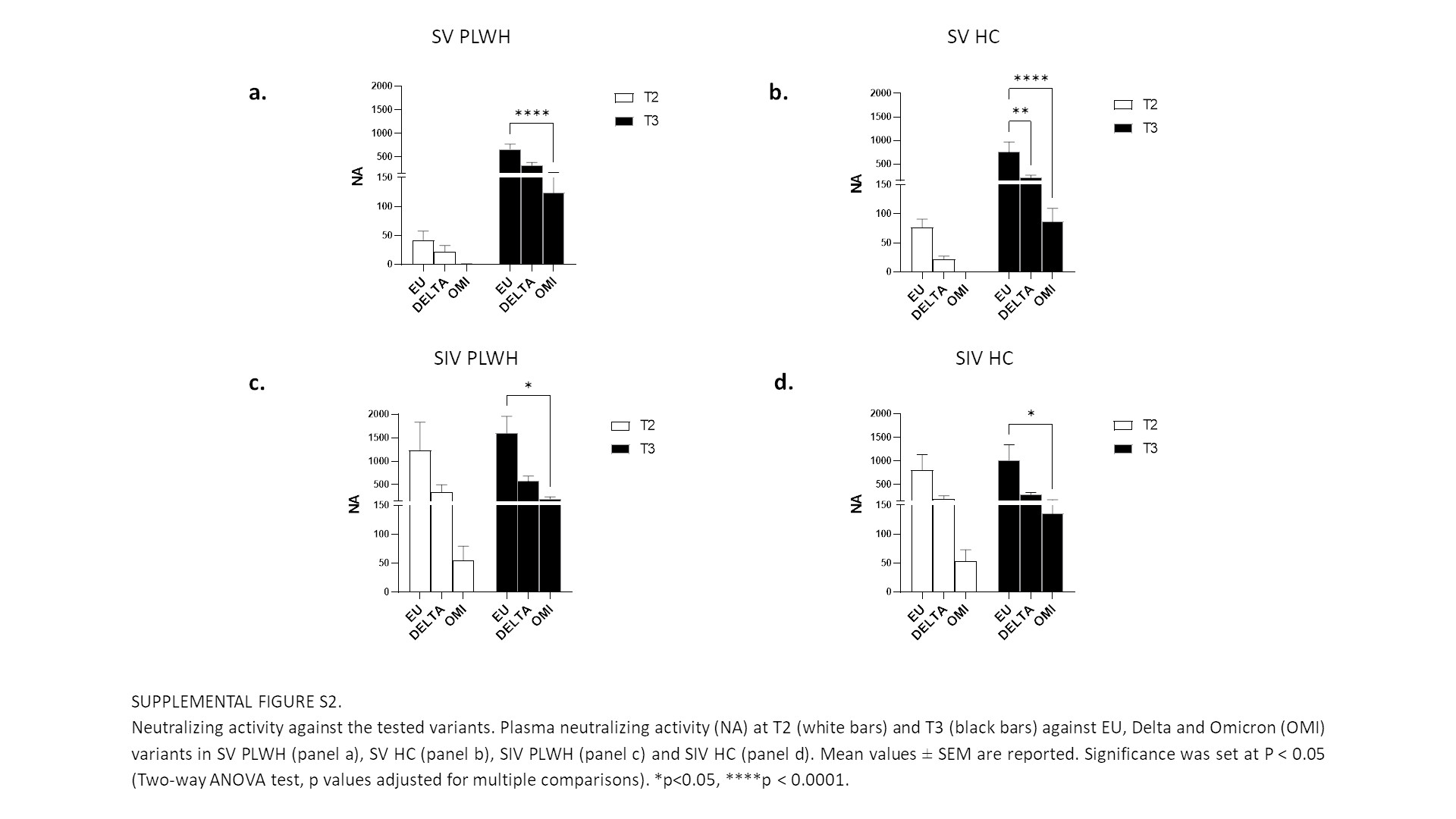

Supplement: Supplementary file 2 [file Image_2.jpg]

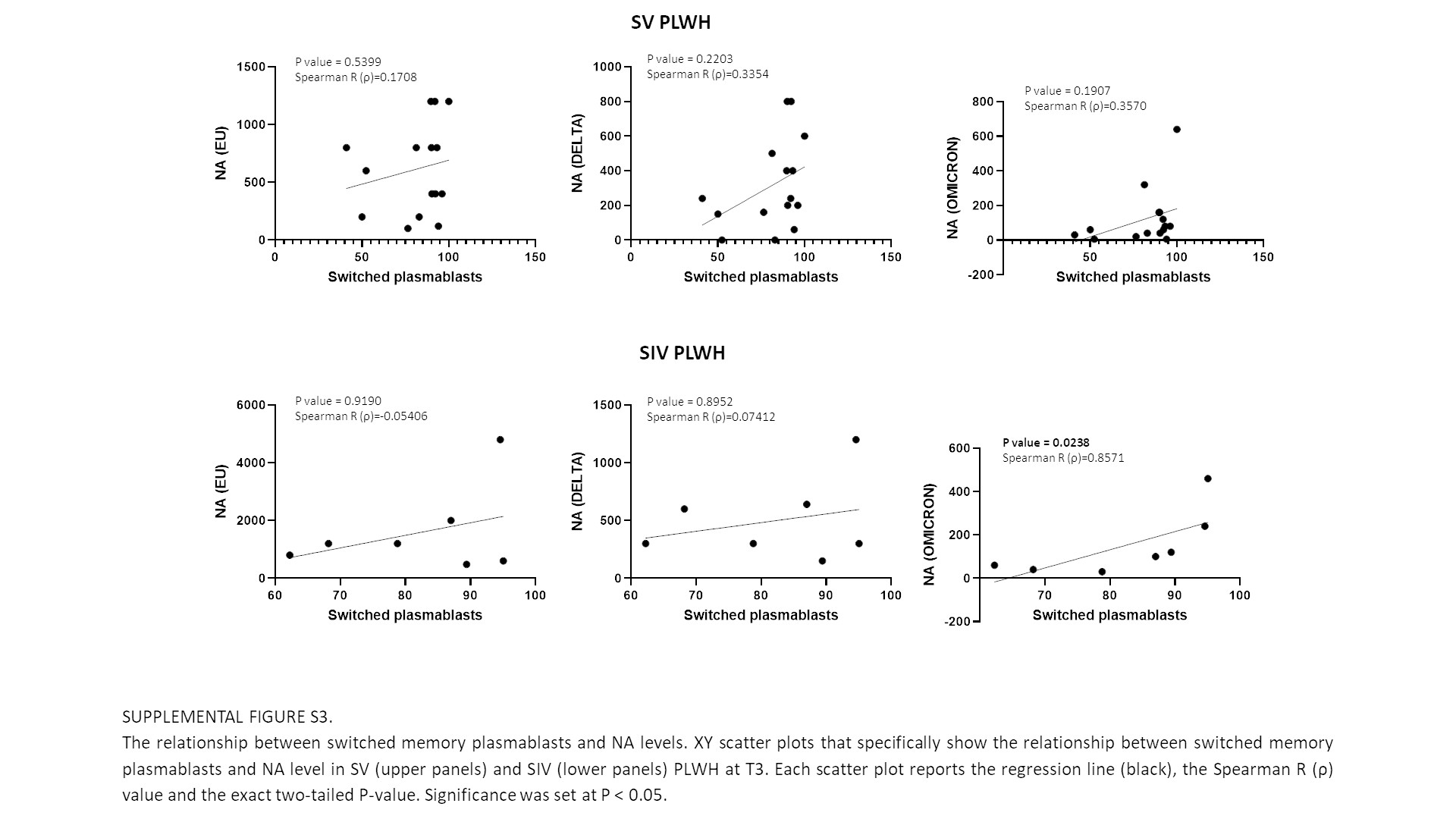

Supplement: Supplementary file 3 [file Image_3.jpg]

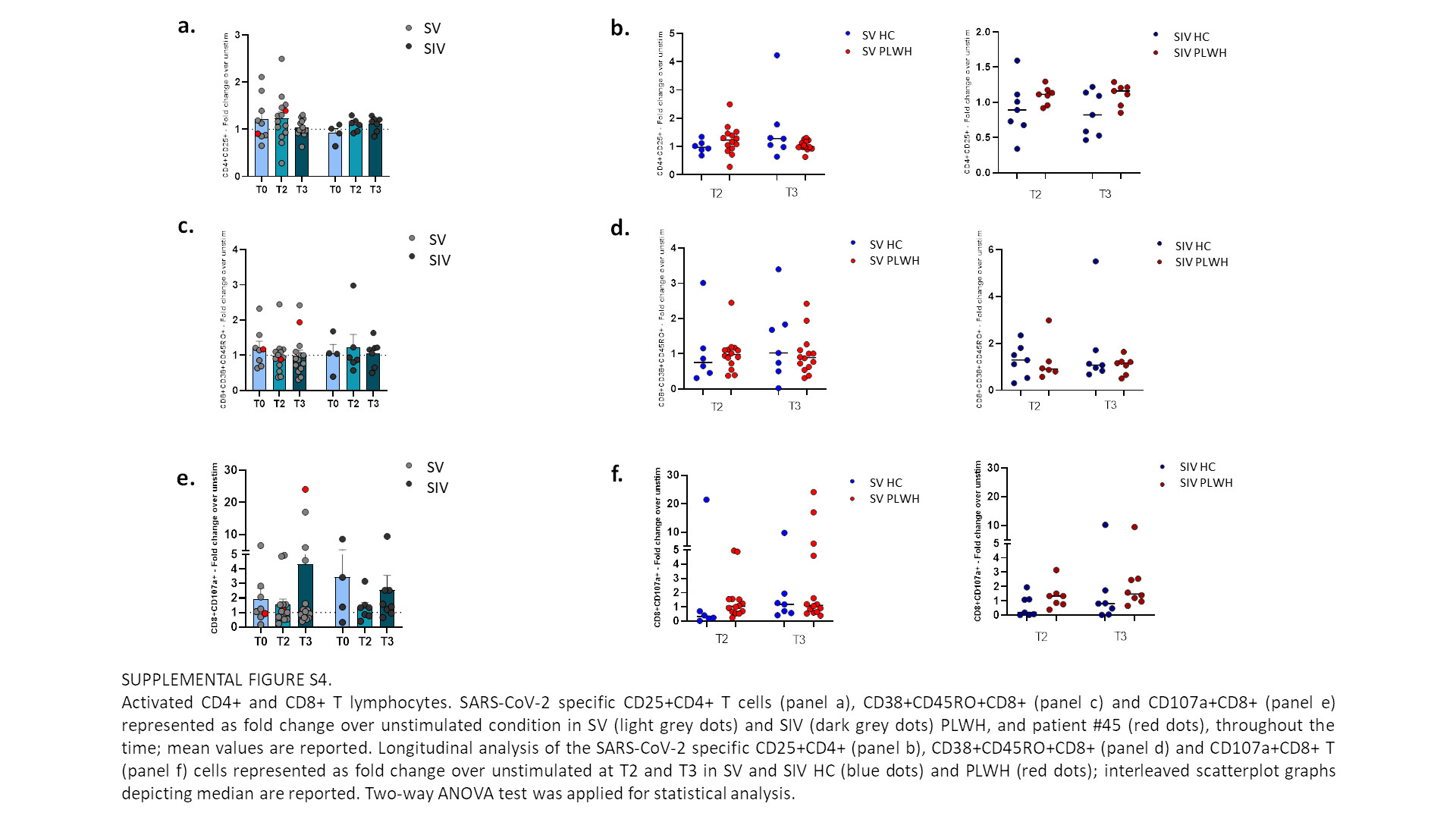

Supplement: Supplementary file 4 [file Image_4.jpg]
