# Supplementary material for: Humoral and cell-mediated immune responses in HIV-vertically infected young patients after three doses of the BNT162b2 mRNA SARS-CoV-2 vaccine
Source: Front Immunol. 2024 Jan 4;14:1301766. doi: 10.3389/fimmu.2023.1301766 (PMC10797701; doi:10.3389/fimmu.2023.1301766)
Supplement: Supplementary file 5 [file Table_1.docx]

Supplemental Table 1. Clinical parameters of patients with low CD4 count and/or detectable viral load at T0 and T3.

| **PATIENT ID** | **Nadir Absolute CD4+ T cell count (cells/mm^3^)** | **Nadir CD4+ T cell percentage** | **Absolute CD4+ T cell count**  **(cells/mm^3^)** | | **CD4+ T cell**  **percentage** | | **HIV-RNA (cp/ml)** | |
| --- | --- | --- | --- | --- | --- | --- | --- | --- |
|  |  |  | **T0** | **T3** | **T0** | **T3** | **T0** | **T3** |
| **#45** | 133 | 5,3 | 206 | 164 | 7,5 | 6,3 | 847 | 506 |
| **#22** | 145 | 13,3 | 235 | 368 | 15,2 | 17 | 314 | <20 |
| **#33** | 317 | 28,2 | 317 | 471 | 28,2 | 34,3 | <20 | <20 |
| **#21** | 338 | 27,3 | 411 | 338 | 27,9 | 27,3 | <20 | <20 |
| **#54** | 190 | 30,1 | 844 | 350 | 26,8 | 29 | 25 | <20 |
| **#27** | 860 | 37,1 | 944 | 1201 | 46,8 | 48,6 | 199 | <20 |
| **#14** | 334 | 21,9 | 894 | 686 | 28,2 | 31,8 | 42 | 35 |
