# Supplementary material for: Humoral and cell-mediated immune responses in HIV-vertically infected young patients after three doses of the BNT162b2 mRNA SARS-CoV-2 vaccine
Source: Front Immunol. 2024 Jan 4;14:1301766. doi: 10.3389/fimmu.2023.1301766 (PMC10797701; doi:10.3389/fimmu.2023.1301766)
Supplement: Supplementary file 6 [file Table_2.docx]

Supplemental Table 2. Significant differences in B cell subset frequencies between timepoints. Significance was set at p<0.05.

|  |  | SARS-CoV-2 specific (*P – Values*) | | |
| --- | --- | --- | --- | --- |
|  |  | T2 vs Baseline | T3 vs Baseline | T3 vs T2 |
| SV PLWH | Plasmablasts | *ns* | *ns* | *ns* |
|  | Switched Memory Plasmablasts | *ns* | *ns* | *ns* |
|  | Double Negative Memory B cells | *ns* | *ns* | *ns* |
|  | Naive B cells | *ns* | *ns* | *ns* |
|  | Switched Memory B cells | *ns* | *ns* | *ns* |
|  | Transitional B cells | *ns* | *ns* | *ns* |
|  | Unswitched Memory B cells | *ns* | *ns* | *ns* |
| SIV PLWH | Plasmablasts | *ns* | *ns* | *ns* |
|  | Switched Memory Plasmablasts | 0.019 | *ns* | *ns* |
|  | Double Negative Memory B cells | *ns* | *ns* | *ns* |
|  | Naive B cells | *ns* | 0.046 | *NS* |
|  | Switched Memory B cells | *ns* | *ns* | *ns* |
|  | Transitional B cells | *ns* | *ns* | *ns* |
|  | Unswitched Memory B cells | *ns* | 0.049 | *ns* |
